# Effect of various flavors in starter diets on growth, behavior, and blood parameters of Holstein calves

**DOI:** 10.1016/j.vas.2024.100388

**Published:** 2024-08-12

**Authors:** Somayeh Fathi, Mohammad Ali Norouzian, Behzad Khorrami, Ali Assadi-Alamouti, Mohammad Reza Bakhtiarizadeh

**Affiliations:** Department of Animals and Poultry Science, College of Aburaihan, University of Tehran, Tehran, Iran

**Keywords:** Flavor, Dairy calves, Weaning age, Behavior, Sodium saccharin

## Abstract

This study investigated the effect of adding various flavors to calf starter concentrates on the performance, behavior, and blood parameters of Holstein dairy calves. The hypothesis was that an optimal flavor enhancer would increase starter intake, improve growth performance, and possibly enable earlier weaning without adverse physiological effects. In Experiment 1, a cafeteria test was conducted with 80 pre-weaned calves to evaluate four synthetic flavor enhancers (salty, sweet, sour, bitter) at levels of 3.3, 6.6, and 9.9 g/kg dry matter (DM)^1^, and a control without a flavor enhancer. Experiment 2 tested higher levels (9.9, 19.8, 29.7 g/kg DM) of the sweet flavor enhancer sodium saccharin in a single cafeteria test with 20 calves. Experiment 3 compared three sweet flavor enhancers (sodium saccharin, stevia, sucralose) at 9.9 g/kg DM in another cafeteria test with 20 calves. After selecting sodium saccharin at 9.9 g/kg DM as the optimal flavor enhancer, Experiment 4 was conducted with 40 calves divided into a treatment group receiving the flavored starter and a control group without flavor. In Experiment 1, sodium saccharin at 9.9 g/kg DM resulted in the highest dry matter intake (DMI)^2^ of 347 g/d, significantly higher than the control (173 g/d). Experiment 2 revealed a quadratic effect with DMI peaking at 9.9 g/kg DM sodium saccharin (201.0 g/d) and declining at higher levels. Experiment 3 confirmed that sodium saccharin outperformed other sweet flavor enhancers, increasing DMI to 350 g/d compared to 251 g/d for the control. In Experiment 4, calves fed the starter concentrate with 9.9 g/kg DM sodium saccharin showed significantly higher DMI (1749 g/d), average daily gain (ADG^3^; 730 g/d), and feed conversion ratio (FCR;^4^ 2.05) compared to the control group (DMI 1470 g/d, ADG 650 g/d, FCR 2.36). Furthermore, calves in the flavored treatment were weaned earlier (59 days) than the control group (64 days). Behavioral observations showed that the sweet flavor treatment increased the time spent on solid feed intake, rumination, and standing behavior. Calves fed the flavored starter had lower levels of cholesterol, triglycerides, and blood urea nitrogen, suggesting improved nutrient utilization. Overall, the results demonstrated the potential benefits of incorporating sodium saccharin as a sweet flavor enhancer in calf starter concentrates, leading to improved feed intake, growth performance, feed efficiency, final body weight (FBW)^5^, and behavioral patterns during the critical pre-weaning and post-weaning periods.

## Highlights

• The incorporation of sodium saccharin as a sweet flavor enhancer at 0.99% dry matter (DM) significantly increased dry matter intake (DMI), average daily gain (ADG), and feed efficiency (FE) in Holstein dairy calves.
• Calves fed the flavored starter concentrate exhibited improved behavioral patterns, such as increased time spent on solid feed intake and rumination, and showed enhanced growth parameters, including body depth, wither height, and hip height.
• The use of sodium saccharin in calf starter concentrates led to earlier weaning, reduced levels of cholesterol, triglycerides, and blood urea nitrogen, suggesting better nutrient utilization and potential cost savings for dairy producers.

## 1 Introduction

Optimal nutrition and feed intake are crucial for ensuring proper growth, development, and overall well-being of dairy calves (Khan et al., 2016; Overton et al., 2013). During the pre-weaning and weaning periods, calves undergo significant physiological and behavioral changes that can influence their feed intake and consequently impact their performance (De Paula Vieira et al., 2012; Silper et al., 2014). One potential strategy to enhance feed intake and promote smooth transitions during this critical phase is the incorporation of flavor compounds in starter concentrates (Miller-Cushon and DeVries, 2015; Nombekela and Murphy, 2022). Flavor perception plays a significant role in the feed acceptance and preference of animals, including dairy calves (Szcześniak et al., 2022). It has been well-established that ruminants possess the ability to detect and respond to basic tastes such as sweet, sour, bitter, and umami (Baumont et al., 2014; Nombekela and Murphy, 2022). These taste perceptions are mediated by gustatory receptors located on the tongue and oral cavity, which trigger neuronal pathways and neuroendocrine responses that regulate feed intake and feeding behavior (Jiang et al., 2022; Webb et al., 2014). Recent studies have investigated the effects of various flavor compounds on feed intake and preference in different livestock species. For instance, the addition of sweeteners like sucrose or saccharin has been shown to enhance feed intake and preference in calves, lambs, and piglets (Cibils et al., 2022; Villalba et al., 2022). Conversely, bitter and sour tastes have been associated with feed aversion and reduced intake in ruminants (Lee et al., 2019; Min et al., 2022). However, the responses to these flavors may depend on the specific compound, concentration, and potential interactions with other dietary components (Schafe et al., 2022; Provenza, 2018).

The inclusion of flavor compounds in calf starter concentrates may not only influence feed intake but also impact other performance parameters such as body weight gain, feed efficiency, and overall growth and development (Ebrahimi et al., 2019; Villalba et al., 2010). Furthermore, the effects of flavor compounds on behavioral patterns such as feeding behavior, rumination, and activity levels have been reported in adult ruminants (Albright and Arave, 1997; Grovum, 1988; Villalba et al., 2015). In addition to performance and behavioral responses, flavor compounds may also modulate physiological parameters, including blood metabolites and hormones related to feed intake regulation and metabolism (Canestrari et al., 2021; Ítavo et al., 2002). For example, research has shown that sweet and umami tastes can stimulate the release of insulin and other anabolic hormones in ruminants, potentially influencing nutrient partitioning and growth (Lee et al., 2019; Min et al., 2022; Nombekela et al., 1994).

While flavor preferences in adult ruminants have been studied, research on how different flavors affect pre-weaned and newly weaned dairy calves is limited. Given the potential benefits of incorporating flavor compounds in calf starter concentrates, further research is needed to understand their effects on various aspects of calf performance, behavior, and physiology. Therefore, the aim of this study was to investigate the effects of different flavors in starter concentrates on the performance, behavioral, and blood parameters of dairy calves, with the goal of developing effective nutritional strategies to optimize calf growth, health, and productivity in dairy production systems.

## 2 Materials and Methods

This study was conducted in four separate experiments at the Saffari Livestock and Agricultural Complex. All experimental procedures received approval from The University of Tehran's Animal Care and Use Committee (Approval No. UT-2023-01). Experiments 1, 2, and 3 were carried out to select the best type and level of flavor enhancer for Holstein pre-weaned calves. After selecting the optimal type and level of flavor enhancer, Experiment 4 was conducted to investigate the effects of using the selected flavor enhancer on the performance characteristics of pre-weaned Holstein calves before and after weaning. Different groups of calves were used for each of the four experiments to avoid any carryover effects. The feed ingredients used in the starter diet formulation and the chemical composition of the starter diet are shown in Table 1.

**Table 1:** Components and Nutritional Composition of the Starter Diet for Holstein Calves.

Components	Percentage (%)
Alfalfa	10
Barley	15
Corn	43.5
Soybean Meal	23
Canola Meal	5
Salt	0.3
Sodium Bicarbonate	1
Calcium Carbonate	1.2
Dicalcium Phosphate	0.5
Vitamins and Minerals	0.5
**Nutritional Components**	
Dry Matter %	89
Protein %	18.5
Fat %	3
Neutral Detergent Fiber (NDF; %)	15
Ash %	7.5
Metabolizable Energy (Mcal/kg)	2.5
Calcium %	0.85
Phosphorus %	0.5

In Experiment 1, to select the best level of flavor enhancer in the starter diet for Holstein pre-weaned calves, a cafeteria test was conducted with 80 male and female calves before weaning. The calves had an average age of 53 ± 2 days and birth weight of 38.1 ± 1.8 kg. Each flavor enhancer consisted of 20 replicates and 5-day periods. The experimental treatments consisted of four synthetic flavor enhancers with salty (potassium chloride; 99% purity, Merck KGaA, Darmstadt, Germany), sweet (sodium saccharin; ≥98% purity, Sigma-Aldrich, St. Louis, MO, USA), sour (sour lemon; natural lemon extract, Iran), and bitter (bitter almond; natural almond extract, Iran) flavors at levels of 3.3, 6.6, and 9.9 g/kg DM, as well as a control without a flavor enhancer. The design was factorial with 20 replicates per treatment. All flavor enhancers were in powder form and were thoroughly mixed with the starter diet. Calves were housed in individual pens (1.2 × 2.4 m) with straw bedding and had access to four buckets with a capacity of approximately 4 liters. The buckets were weighed daily (at 8 a.m. each day) and the feed intake was recorded before being refilled with fresh feed. The location of the buckets was randomly assigned. Throughout the rearing period, calves had ad libitum access to water. Calf behaviors, including solid and liquid feed intake, standing and lying rest, standing and lying rumination, and other activities such as playing, were monitored on the third and fifth days of each test (Silvane Barcelos Carlotto et al., 2007).

In Experiment 2, based on the data obtained from Experiment 1, the sweet flavor enhancer (sodium saccharin) at the 9.9 g/kg DM level, based on DMI during the experimental period and behavioral traits, was determined to be the most effective treatment. To investigate whether higher levels of the sweet flavor enhancer (above 9.9 g/kg DM) could improve DMI, Experiment 2 was designed. For this purpose, a cafeteria test was conducted with calves before weaning, with an average age of 52 ± 3 days and an average birth weight of 38.3 ± 2.1 kg, over 5-day periods. The experimental treatments consisted of the sweet flavor enhancer sodium saccharin at levels of 9.9, 19.8, and 29.7 g/kg DM, as well as a control without a flavor enhancer, mixed with the starter diet. The experiment was conducted in an orthogonal comparison design with four treatments and 20 replicates. Calves were housed in individual pens (1.2 × 2.4 m) with straw bedding. The basal diet consisted of the starter concentrate (Table 1) offered ad libitum, with fresh feed provided daily at 8:00 AM after weighing and recording refusals.

Experiment 3 aimed to identify the most effective sweet flavor enhancer following the determination of the optimal enhancer level. The study employed a completely randomized design involving 20 pre-weaned calves, averaging 52 ± 2 days old and 38.2 ± 2.0 kg at birth. Three synthetic sweet flavor enhancers (sodium saccharin, stevia, and sucralose), approved by European Food Scientific Committee, the European Food Safety Authority, and the U.S. Food and Drug Administration, were tested against a control in a cafeteria-style trial. The enhancers, added at 9.9 g/kg DM based on previous experiments' results, were incorporated into the starter diet and offered alongside the control in separate buckets for a 5-day period, allowing free access to the calves. The buckets were weighed daily (at 8 a.m. each day), and the feed intake was recorded before being refilled with fresh feed. All buckets were washed and disinfected before use. Throughout the rearing period, calves had ad libitum access to water. The calf housing conditions were similar to those in Experiment 1. Calf behaviors were recorded as in Experiment 1.

After selecting the best flavor enhancer and level, Experiment 4 was conducted. In this experiment, 40 male and female Holstein calves (birth weight of 40 ± 2 kg) were used from 30 ± 5 days of age until 10 days after weaning in individual pens. The calves were given a 7-day adaptation period before the experiment began, allowing them to adjust to their new environment. The best flavor enhancer type and level (9.9 g/kg DM sodium saccharin) were mixed with the starter diet and offered to 20 calves (Treatment 1). The remaining 20 calves received the starter diet without a flavor enhancer as the control (Treatment 2). The design was completely randomized with 20 replicates per treatment. All calves had ad libitum access to water. Calves that consumed an average of 1.5 kg of feed per day during a week were weaned. Feed intake was measured daily by subtracting the remaining feed from the initial feed in the bucket using a digital scale. Orts were collected and weighed daily before fresh feed was offered to accurately measure feed intake. Body weight was measured and recorded weekly using a digital livestock scale (Model W300, Irantarazoo, Iran). Feed efficiency (the ratio of body weight gain to feed intake) was also measured. The age at weaning and the average daily weight gain of the calves were recorded. Blood samples were collected at the beginning and end of the experimental period to determine glucose, cholesterol, triglycerides, blood urea nitrogen, beta-hydroxybutyric acid, total protein, creatinine, albumin, alanine aminotransferase (ALT), and aspartate aminotransferase (AST) levels. Samples were taken from the jugular vein using 10 cc vacuum tubes (Far Test, Tehran, Iran) without additives before the evening milk feeding. Samples were centrifuged at 3,000 × g for 15 minutes using a benchtop centrifuge (UNIVERSAL320/320R, HETTICH, Germany) and stored at -20°C until analysis. Serum metabolites were analyzed using commercial kits (Pars Azmoon Co., Tehran, Iran) on an auto-analyzer (Hitachi 917, Roche, Basel, Switzerland).

### 2.1 Statistical Analysis

All statistical analyses were performed using the MIXED and GLM procedures of SAS software (version 9.4, SAS Institute Inc., Cary, NC, USA). Prior to analysis, data were checked for normality using the Shapiro-Wilk test and homogeneity of variances using Levene's test. Initial body weight was included as a covariate in all models to account for weight variability. The models for each experiment included the respective treatment factors as fixed effects: flavor type and level for Experiment 1, sodium saccharin level for Experiment 2, sweetener type for Experiment 3, and treatment (flavored vs. control) for Experiment 4. Calf was included as a random effect in all models. For Experiment 2, orthogonal polynomial contrasts were used to test for linear, quadratic, and cubic effects of sodium saccharin level. For Experiment 4, repeated measures analysis was used for time-dependent variables, with time and the interaction between time and treatment as fixed effects. The significance level was set at P < 0.05 for all experiments. For behavioral data in Experiment 4, a logistic regression model was used within the GLM procedure to analyze the probability of each behavior occurring.

## 3 Results

**Fig. 1:**
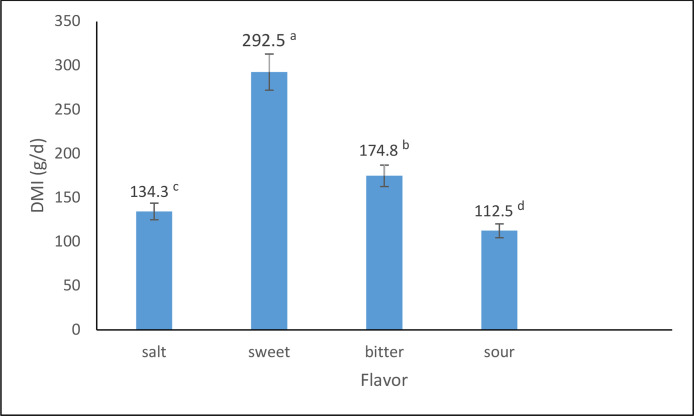
Effect of different flavor on dry matter intake of dairy calves in exp 1.

**Fig. 2:**
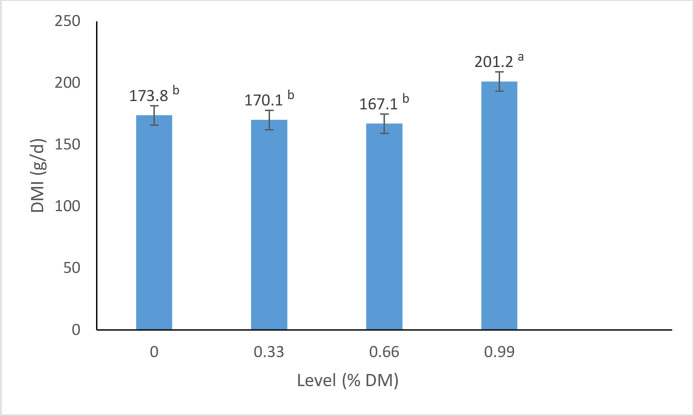
effect of different levels of flavor on dry matter intake of dairy calves in exp 1.

The effects of different flavors and their levels on DMI of dairy calves during the pre-weaning period are presented in Table 1 and Fig. 1, Fig. 2. The results of experiment 1 showed significant differences (P < 0.01) in DMI among the treatments. The addition of the sweet flavor (sodium saccharin) at 9.9 g/kg DM level resulted in the highest DMI (347 g/d), while the sour flavor (sour lemon) at 6.6 g/kg DM level led to the lowest DMI (94 g/d).

**Fig. 3:**
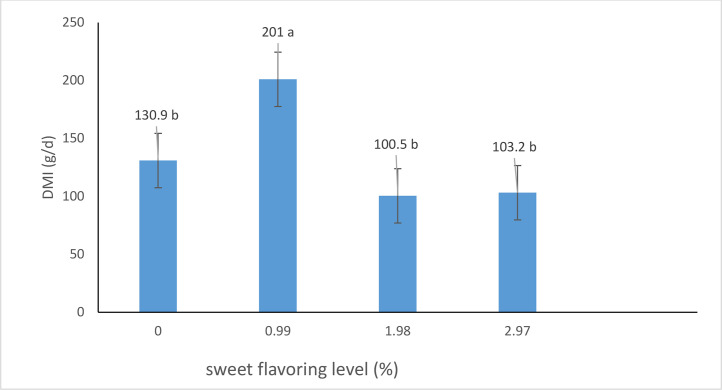
Dry matter intake of calves fed with increased levels of sweet flavoring (g/day) in exp 2.

In experiment 2 (Fig. 3), increasing the level of the sweet flavor (sodium saccharin) above 9.9 g/kg DM resulted in a significant (P < 0.001) decline in DMI, with a quadratic effect (P = 0.25) and a cubic effect (P = 0.0002). The highest DMI (201 g/d) was observed at the 9.9 g/kg DM level of sodium saccharin, while the lowest DMI (100.5 g/d) was recorded at the 19.8 g/kg DM level.

The results of experiment 3 (Fig. 5) revealed that the inclusion of sodium saccharin as a sweet flavor enhancer at 9.9 g/kg DM level significantly (P = 0.025) increased DMI (350 g/d) compared to the control (251g/d) and other flavor enhancers (sucralose and stevia).

In experiment 4 (Table 4), the addition of sodium saccharin (9.9 g/kg DM) as a flavor enhancer in the starter concentrate significantly (P < 0.05) improved the performance of dairy calves during the pre-weaning and post-weaning periods. Calves fed the flavored starter concentrate had higher (P < 0.05) DMI (1749 g/d), ADG (730 g/d), FBW (91.3 kg) and better FCR (2.05) compared to the control group (DMI, 1470 g/d; ADG, 650 g/d; FBW, 85.7 and FCR, 2.36). Furthermore, calves in the flavored treatment group were weaned at an earlier age (59 days) than the control group (64 days).

**Fig. 4:**
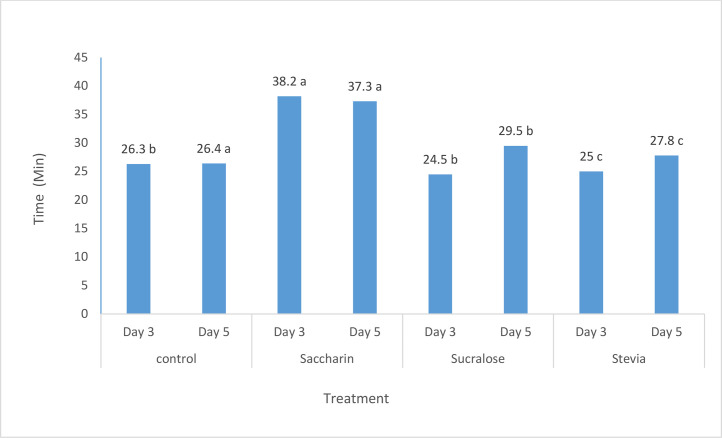
The effect of increasing levels of sweet flavoring on solid feed consumption time of lactating Holstein calves (minutes per day) in exp 2.

The effects of different flavors and their levels on the nutritional and non-nutritive behaviors of dairy calves are presented in Table 3, Table 7 and Figs. 4, and 6. The addition of the sweet flavor (sodium saccharin) at 9.9 g/kg DM level in experiment 1 significantly (P < 0.01) increased the time spent on solid feed intake, water intake, and rumination compared to other flavors and the control (Table 3). Similar trends were observed in experiments 2 and 3, where the inclusion of sodium saccharin at 9.9 g/kg DM level significantly (P < 0.001) increased the time spent on solid feed intake compared to other treatments (Fig. 4, Fig. 6). In experiment 4 (Table 7), calves fed the flavored starter concentrate spent more time (P < 0.01) on solid feed intake, rumination, and standing compared to the control group.

**Fig. 5:**
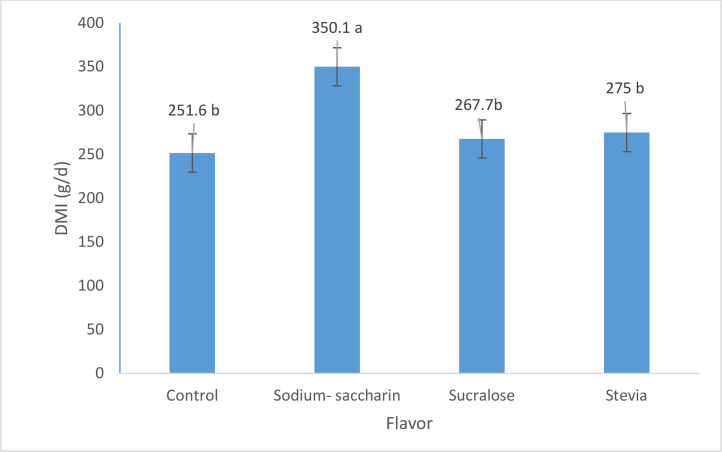
Dry matter intake of calves fed with various sweet flavorings (g/day) in exp 3.

**Fig. 6:**
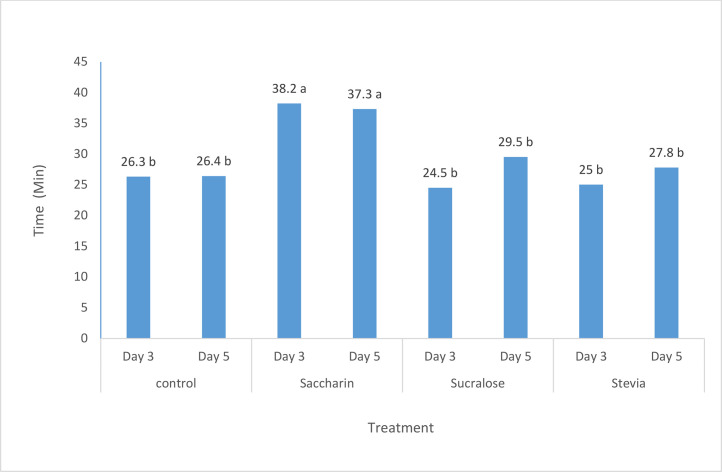
The effect of various sweet flavorings on solid feed consumption time of lactating Holstein calves (minutes per day) in exp 3.

The effects of the experimental diets on blood parameters of dairy calves are shown in Table 5. Calves fed the flavored starter concentrate had significantly (P < 0.05) lower levels of cholesterol, triglycerides, and blood urea nitrogen compared to the control group. However, other blood parameters, including glucose, creatinine, alanine aminotransferase (ALT), aspartate aminotransferase (AST), beta-hydroxybutyric acid, albumin, and total protein, were not significantly different between the two groups.

Table 6 presents the effects of the experimental diets on growth parameters of dairy calves. Calves fed the flavored starter concentrate had significantly (P < 0.05) higher body depth, wither height, and hip height compared to the control group, while other growth parameters (body length, heart girth, hip width, and hip to pin distance) were not significantly different between the two groups.

## 4 Discussion

The findings of this study suggest that the incorporation of flavor enhancers, particularly sweet flavors like sodium saccharin, can significantly modulate the DMI of dairy calves during the critical pre-weaning period. Across multiple experiments, the addition of sodium saccharin as a sweet flavor enhancer consistently resulted in substantial improvements in DMI compared to the control and other flavor treatments.

In experiment 1, the inclusion of sodium saccharin at the 9.9 g/kg DM level led to the highest DMI (347 g/d), significantly outperforming the control (173 g/d) and the sour flavor treatment (sour lemon at 6.6 g/kg DM, which resulted in the lowest DMI of 94 g/d). This observation is consistent with previous research indicating that sweet flavors can stimulate appetite and feed consumption in ruminants (Nombekela et al., 1994b; Solà-Oriol et al., 2011). The mechanism behind this phenomenon can be attributed to the stimulation of gustatory receptors on the tongue and oral cavity by sweet compounds like sodium saccharin. These receptors trigger neuronal pathways and neuroendocrine responses that regulate appetite and feed intake (Torrallardona et al., 2003). Additionally, sweet tastes have been reported to stimulate the release of insulin and other anabolic hormones in ruminants, potentially influencing nutrient partitioning and growth (Nombekela et al., 1994a).

Interestingly, experiment 2 revealed a quadratic and cubic effect on DMI when the level of sodium saccharin was increased beyond the optimal 9.9 g/kg DM level. As the concentration of sodium saccharin increased, DMI initially peaked at 9.9 g/kg DM (201 g/d) but then declined significantly, with the lowest DMI (100 g/d) observed at the highest level of 19.8 g/kg DM. This finding suggests that while moderate levels of sweet flavor enhancers can promote feed intake, excessively high levels may have a counterproductive effect, potentially due to palatability issues or other physiological responses (Karagiannis et al., 2000).

The results of experiment 3 further corroborated the positive impact of sodium saccharin on DMI, as its inclusion at the optimal 9.9 g/kg DM level significantly increased DMI (350 g/d) compared to the control (251 g/d) and other flavor enhancers (sucralose and stevia). This finding highlights the potential superiority of sodium saccharin as a sweet flavor enhancer for promoting feed intake in dairy calves during the pre-weaning period. The observed effects of sweet flavors on DMI can be attributed to their ability to stimulate appetite and feeding motivation. Sweet tastes are generally regarded as highly palatable and attractive to animals, including ruminants (Villalba et al., 2010). The enhanced palatability and preference for the sweet flavor in the starter concentrate may have stimulated feeding behavior and increased the time spent on solid feed intake, as evidenced by the behavioral observations reported in the study.

It is important to note that the responses to different flavors can vary depending on the specific compound, concentration, and potential interactions with other dietary components. For instance, the sour flavor treatment (sour lemon at 6.6 g/kg DM) in experiment 1 resulted in the lowest DMI compared to the control and other flavor treatments. This observation is consistent with previous research indicating that sour and bitter tastes are generally associated with feed aversion and reduced intake in ruminants (Provenza, 1995; Villalba et al., 2010).

Building upon the promising results from the previous experiments, the findings of experiment 4 further reinforce the potential benefits of incorporating sodium saccharin as a sweet flavor enhancer in calf starter concentrates. The addition of sodium saccharin at the optimal level of 9.9 g/kg DM significantly improved various performance parameters of dairy calves during both the pre-weaning and post-weaning periods.

Notably, calves fed the flavored starter concentrate exhibited significantly higher DMI (1749 g/d) compared to the control group (1470 g/d). This substantial increase in feed intake is consistent with the findings from the previous experiments and can be attributed to the palatability-enhancing effects of the sweet flavor, which may have stimulated appetite and feeding motivation (Cibils et al., 2022; Villalba et al., 2022; Albright & Arave, 1997; Ítavo et al., 2002). Furthermore, the improved DMI likely contributed to the observed higher ADG (730 g/d vs. 650 g/d) and better FCR (2.05 vs. 2.36) in the flavored treatment group.

The enhanced growth performance observed in calves fed the flavored starter concentrate can be attributed to the combination of increased nutrient intake, improved palatability, and potential metabolic effects associated with the sweet flavor. Previous research has suggested that sweet flavors may stimulate the release of insulin and other anabolic hormones in ruminants, potentially influencing nutrient partitioning and growth (Lee et al., 2019; Min et al., 2022; Nombekela et al., 1994a). Additionally, the improved FCR in the flavored treatment group suggests more efficient utilization of dietary nutrients for growth and development.

A notable finding from experiment 4 is the earlier weaning age observed in calves fed the flavored starter concentrate (59 days) compared to the control group (64 days). This earlier weaning age is a direct consequence of the higher feed efficiency and growth rates achieved by calves in the flavored treatment group, enabling them to meet the weaning criteria earlier. This finding has practical implications for dairy producers, as earlier weaning can reduce labor and feed costs associated with prolonged milk feeding, while also promoting earlier rumen development and transition to solid feed (Khan et al., 2022).

The behavioral observations across experiments 1, 2, 3, and 4 further elucidate the potential mechanisms underlying the improved performance observed in the flavored treatment groups. The addition of sodium saccharin at the optimal 9.9 g/kg DM level consistently increased the time spent on solid feed intake, rumination, and (in experiment 4) standing behavior. These behaviors are closely linked to feed consumption, nutrient utilization, and overall calf growth and development (Villalba et al., 2022; Yuan et al., 2022).

The increased time spent on solid feed intake and rumination can be attributed to the enhanced palatability and attractiveness of the flavored starter concentrate, which may have stimulated feeding motivation and rumination behavior (Cibils et al., 2022; Villalba et al., 2022; Albright and Arave, 1997; Ítavo et al., 2002). Proper rumination is essential for efficient digestion and nutrient absorption, particularly in ruminants where rumen development is critical during the pre-weaning and post-weaning periods (Khan et al., 2022).

The observed effects on blood parameters, particularly the lower levels of cholesterol, triglycerides, and blood urea nitrogen in calves fed the flavored starter concentrate, may be indicative of improved nutrient utilization and metabolism. These changes could potentially suggest a more efficient utilization of dietary nutrients, leading to lower circulating levels of these metabolites (Lee et al., 2019; Min et al. 2022; Grovum and Chapman, 1988). However, it is important to note that other blood parameters, such as glucose, creatinine, liver enzymes (ALT and AST), beta-hydroxybutyric acid, albumin, and total protein, were not significantly different between the two groups, suggesting that the flavor enhancer did not adversely affect these key metabolic markers.

The observed improvements in growth parameters, specifically body depth, wither height, and hip height, in calves fed the flavored starter concentrate further reinforce the positive impact of the flavor enhancer on overall growth and development. Proper skeletal development is crucial for ensuring optimal conformation and longevity of dairy cows, and these findings suggest that the enhanced nutrient intake and utilization supported better skeletal growth in the flavored treatment group (Khan et al., 2022; Lee et al., 2019; Grovum, 1988).

While the results of this study provide strong evidence for the benefits of incorporating sodium saccharin as a sweet flavor enhancer in calf starter concentrates, it is important to acknowledge the potential role of other flavor compounds in modulating feed intake and performance in dairy calves (Table 2). For instance, the results of experiment 1 showed that the sour flavor (sour lemon) at the 6.6 g/kg DM level led to the lowest DMI compared to the control and other flavor treatments. This observation is consistent with previous research indicating that sour and bitter tastes are generally associated with feed aversion and reduced intake in ruminants (Cibils et al., 2022; Karagiannis et al., 2000; Provenza, 1995).

**Table 2:** effect of different treatments on dry matter intake of dairy calves in experiment 1.

	Control		salt		sweet			bitter			Sour		SEM			P value			
		0	0.33	0.66	0.99	0	0.33	0.66	0.99	0	0.33	0.66	0.99	0	0.33	0.66	0.99	0	0.3
DMI (g/d)	173.0 ^d^	141.3^de^	135.2^e^	125.6^e^	135.2^e^	242.5^c^	283.7^b^	296.0^b^	347.8^a^	186.5^d^	168.5 ^d^	152.1^d^	192.0 ^d^	125.0^e^	101.1^f^	94.1^f^	129.7^e^	10.25	<0.01

**Table 3:** The effect of using different flavorings on nutritional and non-nutritive behaviors of lactating Holstein calves (minutes per day) in exp 1.

Items		Salt	Sweet	Bitter	Sour	SE	p-value
Solid feed	Day 3	65^b^	103^a^	83^a^	73^a^	5.88	0.009
	Day 5	56^c^	112^a^	89^b^	66^c^	4.72	0.0001
Water intake	Day 3	7^a^	4^b^	4^b^	4^b^	0.47	0.004
	Day 5	8^a^	5^b^	4b^c^	3^c^	0.38	<0.0001
Rumination	Day 3	71^b^	120^a^	95^c^	85^bc^	6.42	0.004
	Day 5	82^b^	185^a^	113^b^	93^b^	10.47	0.0005
Stand	Day 3	85^a^	140^ab^	110^a^	115^a^	14.92	0.15
	Day 5	105^a^	95^a^	98^a^	110^a^	9.83	0.70
Laid down	Day 3	474^a^	318^b^	398^a^	403^a^	25.12	0.01
	Day 5	439^a^	293^b^	381^a^	421^a^	21.36	0.005
Idleness	Day 3	18^b^	35^a^	30^ab^	40^a^	4.74	0.05
	Day 5	30^a^	30^a^	35^a^	27^a^	3.96a	0.57a

**Table 4:** The effect of experimental diets on the performance and weaning age of lactating Holstein calves (g/day) in exp 4.

Trait	Treatment		SE	P-value			
	control	flavor		trt	sex	period	Period *Treat
DMI(g/d)							
Total	1470.15^b^	1749.39^a^	60.55	0.002	0.02	<0.0001	0.003
Pre-weaning	683.61	749.07	66.75	NA	NA	NA	NA
Post-weaning	2256.69	2749.71	87.26	NA	NA	NA	NA
ADG (g/d)							
Total	650^b^	730^a^	0.02	0.04	0.08	<.0001	0.51
Pre-weaning	370	480	0.04	NA	NA	NA	NA
Post-weaning	930	980	0.04	NA	NA	NA	NA
FCR							
Total	0.44^a^	0.41^b^	0.10	0.03	0.33	<0.0001	0.89
Pre-weaning	0.54	0.64	0.11	NA	NA	NA	NA
Post-weaning	0.41	0.35	0.11	NA	NA	NA	NA
Final BW (kg)							
Total	85.70^b^	91.30^a^	0.92	0.001	0.02	<0.0001	<0.0001
Pre-weaning	81.10	85.50	0.93	NA	NA	NA	NA
Post-weaning	90.25	97.10	0.93	NA	NA	NA	NA
Weaning age (d)	64.45^a^	59.30^b^	0.65	<.0001	0.36	NA	NA

**Table 5-:** Effect of experimental diets on blood parameters of lactating Holstein calves.

Parameter	Treatment			
	control	flavor	SE	P value
Glucose (mg/dl)	90.94^a^	96.93^a^	2.81	0.15
Cholesterol (mg/dl)	115.04^a^	94.89^b^	5.48	0.02
Triglyceride (mg/dl)	30.38^a^	19.47^b^	3.45	0.04
Blood urea nitrogen (mg/dl)	24.85^a^	19.50^b^	1.66	0.04
Creatinine (mg/dl)	0.86^a^	0.83^a^	0.04	0.60
ALT (IU/l)	70.87^a^	63.61^a^	4.64	0.28
AST (IU/l)	17.35^a^	16.82^a^	0.77	0.64
Beta hydroxy butyric acid (mmol/l)	0.30^a^	0.26^a^	0.03	0.56
Albumin (g/lit)	2.86^a^	2.94^a^	0.07	0.47
Total protein (g/lit)	6.96^a^	6.71^a^	0.25	0.49

**Table 6:** The effect of experimental diets on growth factors of lactating Holstein calves.

Trait	Treatment		SE	P-value
	control	flavor		
Body Length, cm	95.89^a^	97.60^a^	0.78	0.13
Body depth, cm	120.05^b^	124.44^a^	1.45	0.04
Heart girth, cm	104.24^a^	104.75^a^	0.73	0.63
Wither height, cm	94.60^b^	96.89^a^	0.41	0.001
Hip height, cm	98.21^b^	100.36^a^	0.55	0.01
Hip width, cm	18.03^a^	19.04^a^	0.38	0.07
Hip to Pin distance, cm	28.93^a^	29.39^a^	0.32	0.33

**Table 7:** The effect of experimental diets on nutritional and non-nutritive behaviors of lactating Holstein calves (minutes per day).

Items	Treatment		SE	p-value
	Flavors	Control		
Solid feed	141.29^a^	121.85^b^	0.05	0.006
Water intake	4.24^a^	4.56^a^	0.09	0.43
Rumination	188.70^a^	162.01^a^	0.07	0.06
Stand	106.15^a^	89.26^b^	0.08	0.05
Laid down	242.74^a^	295.70^b^	0.07	0.009
Idleness	27.28^a^	31.34^a^	0.16	0.39

## 5 Conclusion

In summary, the findings of this study provide strong evidence for the potential benefits of incorporating sodium saccharin as a sweet flavor enhancer in calf starter concentrates. The inclusion of sodium saccharin at the optimal level of 9.9 g/kg DM significantly improved various performance parameters, including dry matter intake, average daily gain, feed efficiency, and behavioral patterns, during the critical pre-weaning and post-weaning periods. The earlier weaning age observed in the flavored treatment group also has practical implications for dairy producers, potentially reducing feed and labor costs.

## CRediT authorship contribution statement

**Somayeh Fathi:** Project administration, Methodology, Investigation, Data curation. **Mohammad Ali Norouzian:** Writing – review & editing, Writing – original draft, Supervision, Investigation, Conceptualization. **Behzad Khorrami:** Validation, Supervision. **Ali Assadi-Alamouti:** Supervision, Project administration, Methodology, Conceptualization. **Mohammad Reza Bakhtiarizadeh:** Formal analysis, Data curation.

## Declaration of competing interest

None of the authors have any conflict of interest to declare.
